# Vestibular compensation: the neuro-otologist’s best friend

**DOI:** 10.1007/s00415-015-7903-4

**Published:** 2016-04-15

**Authors:** Michel Lacour, Christoph Helmchen, Pierre-Paul Vidal

**Affiliations:** Université Aix-Marseille/CNRS, UMR 7260, Fédération de Recherche 3C, Centre de St Charles, 3 Place Victor Hugo, 13331 Marseille Cedex 03, France; Department of Neurology, University Hospitals Schleswig–Holstein, University of Lübeck, Ratzeburger Allee 160, 23538 Lübeck, Germany; Université Paris Descartes/CNRS, UMR-MD-SSA, COGNAC-G (COGNition and Action Group), 45 Rue des Saints Pères, 75270 Paris Cedex 06, France; 21 Impasse des Vertus, 13710 Fuveau, France

**Keywords:** Unilateral vestibular loss, Vestibular compensation, Static deficits recovery, Dynamic deficits recovery, Animal models, Human brain imaging

## Abstract

Why vestibular compensation (VC) after an acute unilateral vestibular loss is the neuro-otologist’s best friend is the question at the heart of this paper. The different plasticity mechanisms underlying VC are first reviewed, and the authors present thereafter the dual concept of vestibulo-centric versus distributed learning processes to explain the compensation of deficits resulting from the static versus dynamic vestibular imbalance. The main challenges for the plastic events occurring in the vestibular nuclei (VN) during a post-lesion critical period are neural protection, structural reorganization and rebalance of VN activity on both sides. Data from animal models show that modulation of the ipsilesional VN activity by the contralateral drive substitutes for the normal push–pull mechanism. On the other hand, sensory and behavioural substitutions are the main mechanisms implicated in the recovery of the dynamic functions. These newly elaborated sensorimotor reorganizations are vicarious idiosyncratic strategies implicating the VN and multisensory brain regions. Imaging studies in unilateral vestibular loss patients show the implication of a large neuronal network (VN, commissural pathways, vestibulo-cerebellum, thalamus, temporoparietal cortex, hippocampus, somatosensory and visual cortical areas). Changes in gray matter volume in these multisensory brain regions are structural changes supporting the sensory substitution mechanisms of VC. Finally, the authors summarize the two ways to improve VC in humans (neuropharmacology and vestibular rehabilitation therapy), and they conclude that VC would follow a “top-down” strategy in patients with acute vestibular lesions. Future challenges to understand VC are proposed.

## Vestibular compensation: a model of neuronal plasticity

The vestibular system contributes to reflex generation for posture [1] and oculomotor [2] control, and interacts with high-level cognitive processes including spacial perception [3], spacial navigation [4] and body representation [5]. Alteration of the vestibular inputs as a consequence of ageing, head trauma, ototoxic drugs or vestibular pathologies has dramatic consequences particularly on balance control and gaze stability, both impaired functions that have a strong impact on the patients’ quality of life [6]. Indeed, vertigo and dizziness, and their associated neurovegetative symptoms nausea and vomiting, are extremely disabling for vestibular loss patients who exhibit increased anxiety and depression compared to healthy subjects [7]. Together with the physical disability and the psychological stress, the socio-professional consequences that accompany vestibular damage are major in many cases (stop working, social isolation) [8].

Fortunately, acute vestibular syndrome is ameliorated over weeks and months in both animals and humans through the process of vestibular compensation (VC). Indeed, there is a spontaneous functional recovery after damages to the vestibular system, one of the best documented post-lesional neuronal and behavioural plasticity ([9–12] for reviews). For this reason, VC can be seen as the neuro-otologist’s best friend: the intrinsic plasticity of the nervous system to reorganize is able to overcome the damages of the peripheral vestibular system. However, the time course of recovery shows strong inter-individual variations and the final level of recovery as well. Intrinsic and extrinsic factors that modulate the VC process appear responsible for poorly compensated vestibular loss patients.

**Fig. 1:**
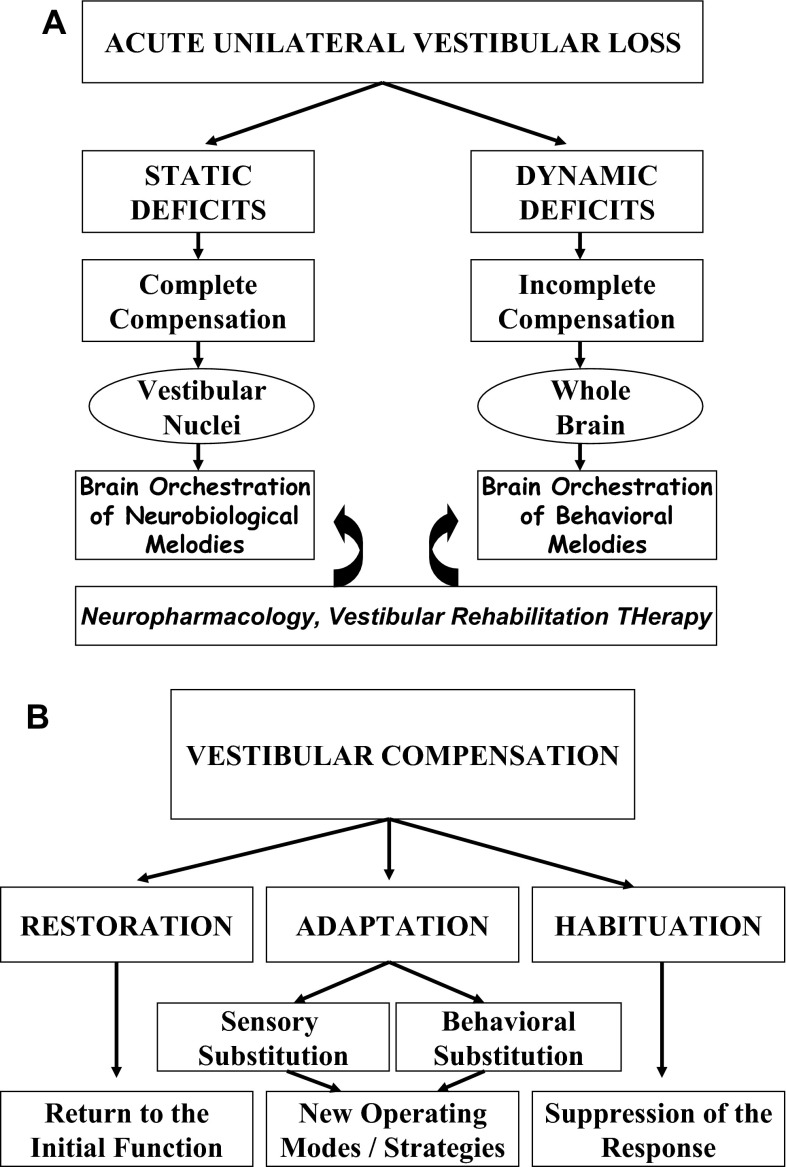
Vestibular syndrome and recovery mechanisms in the compensation of acute unilateral vestibular loss. **a** The static ocular motor, postural and perceptive deficits are completely compensated by a rebalance of activity within the vestibular nuclei (VN) complexes on both sides. The orchestration of various neurobiological responses (or melodies) to VN deafferentation, depending on vestibular aetiology, is responsible for this re-balanced spontaneous firing between the VN on both sides. In contrast, the dynamic deficits (impaired vestibulo-ocular reflex and balance control in challenging conditions) are incompletely compensated. The whole brain functionally reorganizes and expresses new strategies (orchestration of behavioural responses or melodies) depending on the patients themselves. Neuropharmacology as well as vestibular rehabilitation therapy can alter the recovery of both the static and dynamic functions. **b** Main concepts explaining vestibular compensation: restoration, adaptation (sensory and behavioural substitutions) and habituation

The VC process is based on several concepts called Restoration, Habituation and Adaptation that are improperly used in many cases ([13, 14] for reviews) (Fig. 1b). Restoration means that the lost function is recovered with the same nervous connectivity as before the vestibular damage. The intrinsic capacity of peripheral vestibular synapses to regenerate was demonstrated in vitro [15]. The only indirect indication of such a structural repair in humans has been shown in some patients diagnosed with acute unilateral vestibular neuritis during the video head impulse test (vHIT) [16]. A full restoration of horizontal canal function was observed some months later, as shown in these patients by a vestibulo-ocular reflex (VOR) gain close to unity during unpredictable head turns towards the lesion side. Such VOR recovery can be explained by regeneration of peripheral sensory hair cells, or sprouting of new afferent terminals from remaining fibres in the vestibular nerve, or increased synaptic weight of remaining vestibular inputs. Habituation is aimed at reducing progressively the vestibular lesion-induced asymmetry at the peripheral or central levels by repetition of the triggering signals. Even though VOR habituation has been used as a paradigm to study plasticity in the vestibular system, this mechanism does not play a significant role in the VC process. By contrast, adaptation is a powerful recovery mechanism. It is referred in the literature as two separate entities, called sensory substitution and behavioural substitution [12–14]. The lost functions are not restored but replaced by new operating modes using either other sensory cues or newly elaborated motor strategies. Sensory substitution plays a key role since vestibular functions are multisensory determined and need the integration of vestibular, visual, and somatosensory cues [17], which constitute potential sources of possible sensory reweighting. Behavioural substitution is based on the distributed property of the CNS to control the vestibular functions, several neuronal networks in the brain being able to reorganize functionally by learning, and to mimic the lost dynamic vestibular functions. A nice illustration is the covert saccade during head impulse test, which is a saccadic substitution of the normally slow phase eye movement, aimed at preventing oscillopsia during head rotation towards the lesion side [18].

## The static and the dynamic vestibular deficits are recovered differently

The vestibular syndrome after a unilateral vestibular loss is made of both static symptoms, observed in a stationary subject, and dynamic symptoms seen only when the patient moves his/her head or his/her whole body in space [12] (Fig. 1a) The static deficits constitute the ocular-tilt reaction, that is, a combination of ocular motor signs (spontaneous vestibular nystagmus, skew deviation, eye cyclotorsion), postural signs (head and body tilt to the lesion side), and perceptive signs (vertigo, tilt of the subjective visual vertical). The static syndrome results from the combination of the effects of the horizontal canal and utricular lesions, and is more accentuated in the frontal than in the sagittal plane [19]. This static syndrome is fully compensated in unilateral vestibular loss patients with a longer time constant (3 months for the postural and ocular motor deficits, up to 1 year for the perception of verticality) [11, 12, 20], compared to animal models (1 week in the rat, mice and guinea pig, 6 weeks in the cat). In contrast, the dynamic deficits remain poorly compensated and are exhibited over a longer time period [11, 12, 20]. That is the case for the drop in gain and phase shift of the VOR, the reduction of the time constant of the VOR, the impaired balance control in challenging conditions. In most of the cases, the VOR recovers poorly at both low and high frequencies. The reason why, however, such patients do not complain of oscillopsia and instability during fast head rotation is their capacity to elaborate new eye–head coordination strategies and to use other triggering signals (the saccadic substitution, see above). VC therefore includes a fast vestibulo-centric static process, and a longer term, dynamic, distributed learning process. The dual concept of brain orchestration of “neurobiological signatures, or melodies” and “behavioural signatures, or melodies” has been proposed to explain the recovery of the static and dynamic functions, respectively [21]. It will be shown in the following that the former depends on the vestibular aetiology, while the latter depends on the patients themselves.

### Recovery of the static deficits

Acute unilateral vestibular deafferentation induces a strong imbalance in the resting discharge of the VN complexes on both sides [22, 23]. A drop of the spontaneous firing rate and sensitivity of the type I VN neurons is observed in the ipsilesional medial VN, and an increased inhibitory drive from the contralesional side, through the inhibitory commissural pathways, still enhances this imbalance. The Bechterew phenomena, that is, the mirror image of the static symptoms when the intact labyrinth is destroyed some time after the initial lesion, is a powerful proof that plastic events occur in the deafferented VN complex and rebalance the resting discharge on both sides.

**Fig. 2:**
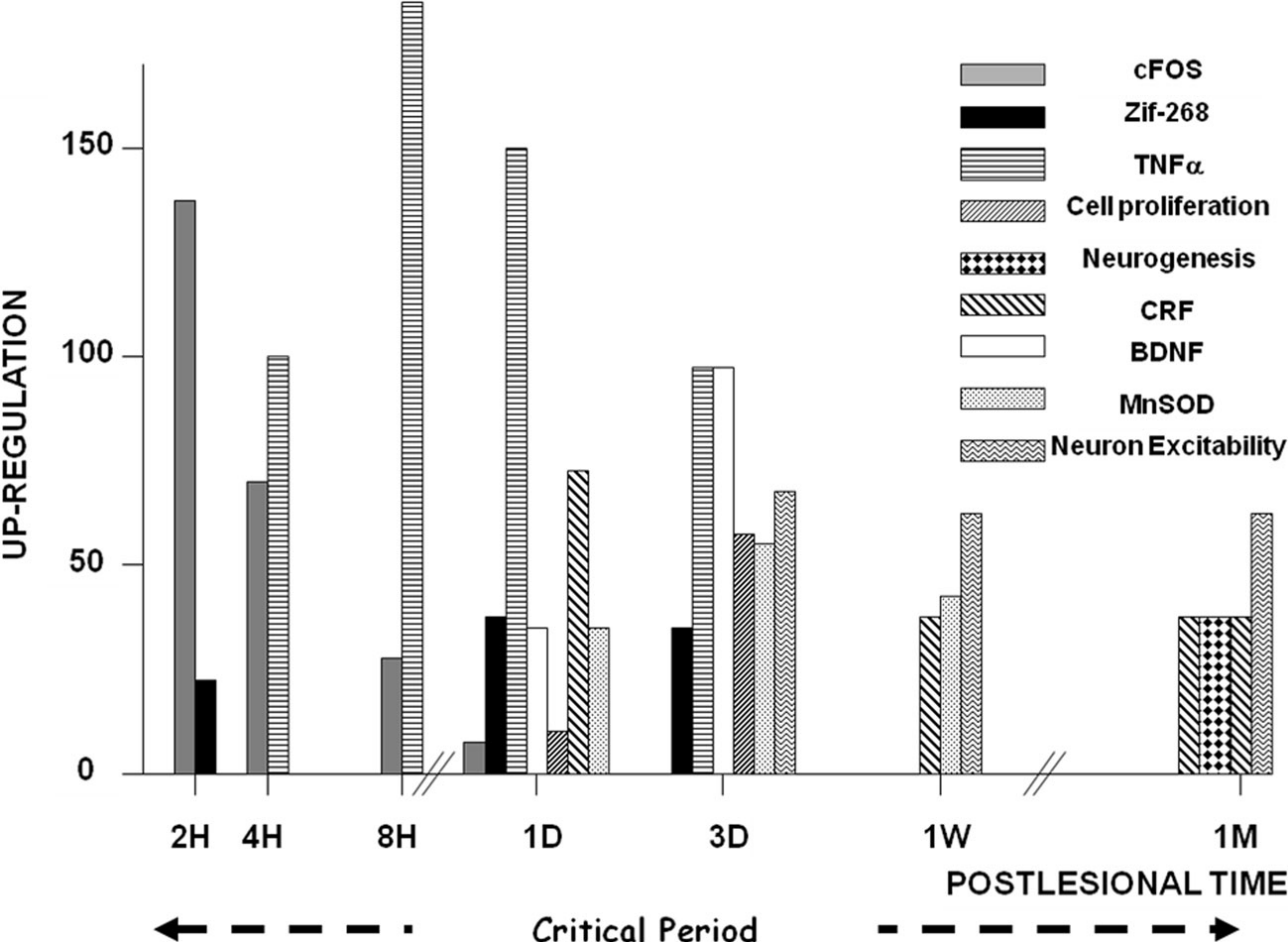
Molecular and cellular mechanisms involved in vestibular compensation. The figure illustrates the plastic events occurring in the ipsilesional vestibular nuclei (VN) complex after acute unilateral vestibular loss. Up-regulation of the immediate early genes Fos and Zif-268 is found in the very first hours and days following the deafferentation, which induces a cascade of plastic events. Neurotrophic (BDNF) and neuroprotective (MnSOD) factors, markers of inflammation (TNFα), markers of the stress axis activation (corticotrophin-releasing factor: CRF) are up-regulated. Cell proliferation is seen very early (glial reaction, astrogenesis and neurogenesis) and is followed later on by cell differentiation leading particularly to newborn GABAergic neurons. Changes in intrinsic excitability of the VN neurons are observed also during the first post-lesional month, which could constitute an opportunity window, that is, a critical period for structural and functional reorganization in the ipsilesional VN complex. Up-regulations after vestibular lesion are expressed in percent of the basal level recorded in intact controls. Adapted from [13, 24, 26–28, 80]

Immediate early genes (Fos, June, Zif-268: see [24]) are up-regulated within a few hours after the lesion. They induce a cascade of molecular and cellular events in the following hours and days. A microglial response is apparent as early as 1 day and persists for several weeks in the rat [25] as well as in the cat [26, 27] models. It correlates with increased levels of inflammation markers (TNFα), of neuroprotective (MnSOD, NFkB), and of neurotrophic (BDNF) factors [26, 28]. Astroglia also show an intense activation within the first 3 days. This intense cellular activity is supported by modifications of the transcriptomes in the VN complexes [29, 30]. Up-regulation of proteins implicated in the cellular metabolism is therefore not surprising [31]. Recent investigations aimed at visualizing the relative changes of glucose metabolism (rCGM) showed in the acute stage a significant asymmetry in the VN complexes and related structures (vestibulo-cerebellum, thalamus, vestibular cortex, hippocampus and amygdale), followed during the time course of recovery by re-balanced rCGM in these structures [32] (see “Imaging studies in unilateral vestibular loss patients”). Proteins involved in axonal growth and guidance are also up-regulated [31], suggesting a structural reorganization of the network within the nuclei during compensation. A strong reactive cell proliferation occurs in the ipsilesional VN; most of the newborn cells survive and differentiate into glial cells and neurons with a GABAergic phenotype [27, 33, 34]. It appears therefore that neural protection and structural reorganization are the main challenges of the post-lesional events occurring in the ipsilesional VN complex, during an early opportunity window or critical period for optimal behavioural recovery (Fig. 2).

How is the spontaneous activity of the ipsilesional VN complex restored? This is a key question since all the static deficits (ocular-tilt reaction, vertigo) are explained by the asymmetrical resting discharges between the two sides, and their recovery by the return to symmetrical firing rates on both sides. The sensitivity of ipsilesional VN neurons to inhibitory neurotransmitters like GABA and glycine is decreased so that the inhibitory drive from the contralesional side is reduced [35, 36]. Decrease in GABA_A_ sensitivity of the neurons could be explained by modifications of extrasynaptic receptors [37]. Return to normal GABA_A_ sensitivity is observed within a few days, while GABA_B_ receptors remain down-regulated [38]. The role of the excitatory amino acid receptors still remains unclear [39], even though remarkable plasticity is observed at the synapse of post-synaptic type B neurons, with long-term potentiation (LTP) or long-term depression (LTD) depending on the polarization level of the cell or of the firing pattern of the pre-synaptic fibre [40]. The long-burst protocol used to induce LTP or LTD under physiological conditions induces also a long-term potentiation of intrinsic excitability in most of the type A and half the type B neurons by activation of mGluR1 receptors [41]. Increased intrinsic excitability of ipsilesional VN cells [39] combined with decreased sensitivity to inhibitory neurotransmitters constitute a physiological response able to counteract both initial disfacilitation and increased commissural inhibition from the intact side, and to restore a balanced activity between the two sides. This combination of cellular mechanisms is responsible for the recovery of the static vestibular functions. The underlying molecular mechanisms are less clear but obvious candidates are the Ca^++^-dependent potassium channels, like the BK channels [42] that play a key role in intrinsic excitability through various phosphorylation sites [43]. Conductance is decreased by protein-kinase C-mediated phosphorylations, and is increased by the activated calmodulin-kinase II. Increased intrinsic excitability still persists on the long term to compensate the loss of the afferent excitatory vestibular drive. Convergence of multisensory inputs to the VN complex could explain this permanent change [44]. Modifications in the proportion of type A and type B neurons in the ipsilesional and contralesional VN complexes are additional arguments for the compensation of the static deficits. Reinforcement of the “B-like” properties on the contralesional side and of the “A-like” properties on the ipsilesional side was found [45–47]. In the compensated stage, the tonic activity of the ipsilesional VN neurons is modulated by the contralesional drive, a functional reorganization substituting to the normal push–pull mechanism between the two sides.

### Recovery of the dynamic deficits

Electrophysiological and behavioural investigations in animal models support the general view that the full compensation of the static ocular motor and postural deficits results from the fast rebalance of spontaneous resting discharges in the VN complexes on both sides, while the dynamic symptoms improve more slowly and never fully compensate [12, 26]. In addition, it is noteworthy that the recovery of the dynamic symptoms is less dependent of the recovery of the static symptoms and requires that the brain promotes vicarious processes and new operating modes [48].

Synaptic remodelling (neurogenesis, astrogenesis, synaptogenesis) is a potentially structural mechanism operating on the long term. The cell proliferation observed in the cat model of UVN, peaking at three days, and the cell differentiation observed 1–3 months later are blocked under antimitotic drugs treatment [49], and are strongly altered with chronic infusion of the GABA_A_ receptor agonist muscimol in the VN [50]. These studies show that posturo-locomotor recovery is dramatically impaired in such conditions, whereas the fastest behavioural recovery is observed with the VN infused with GABA_B_ receptor antagonist. It must be mentioned, however, that cell proliferation and cell differentiation are not seen after unilateral labyrinthectomy or reversible blockade of the afferent vestibular drive by tetrodotoxin [33, 34], indicating that neurogenesis within the VN networks strongly depends on vestibular aetiology. This structural remodelling is expected in patients with acute and sudden near total vestibular loss (vestibular neuritis for example) [51].

**Fig. 3:**
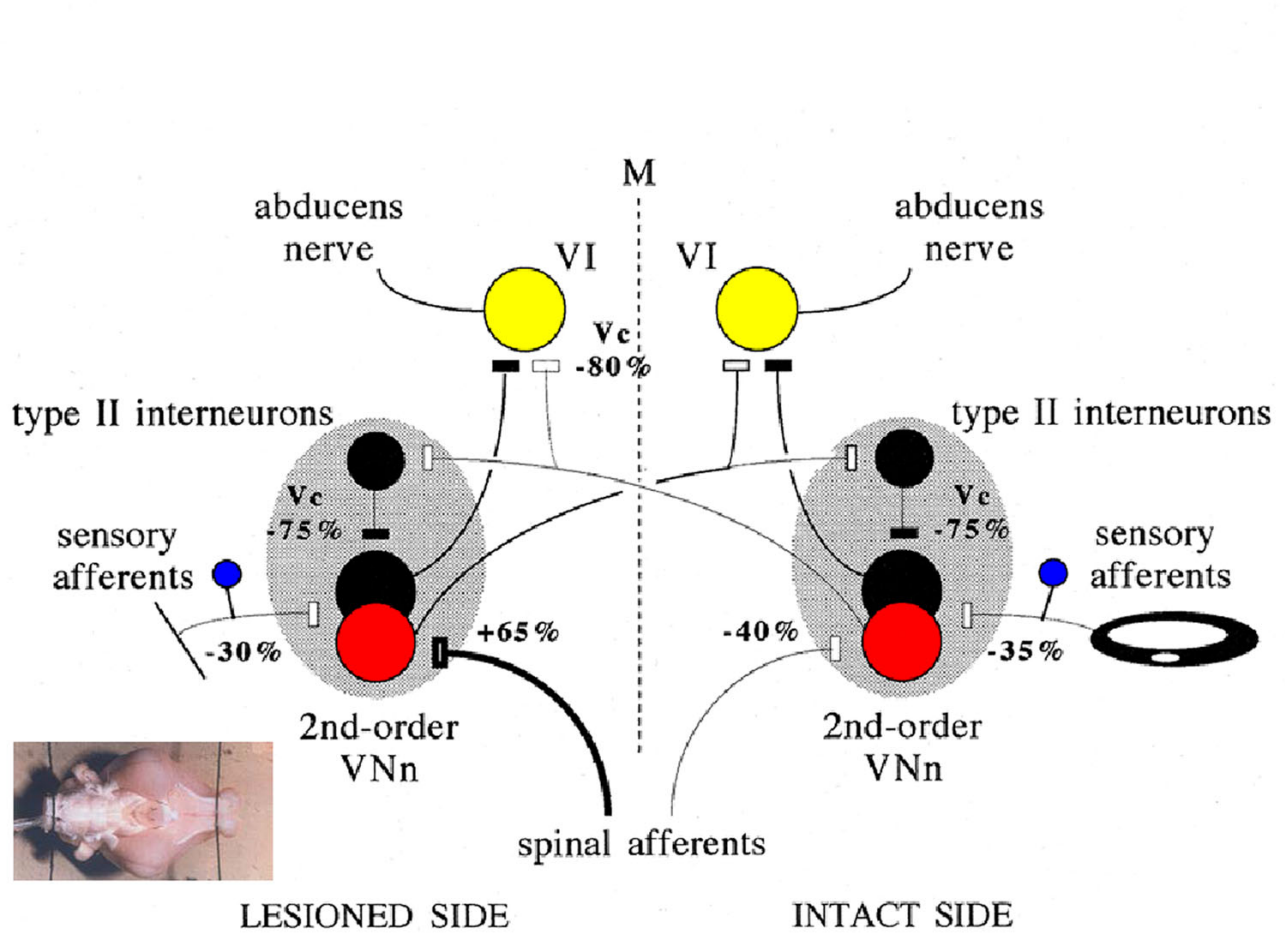
Plastic changes recorded in vitro that may underlie vestibular compensation in vivo. The figure (issued from [47]) shows the putative changes of synaptic efficacy, which have taken place in the vestibular-related pathways of compensated guinea pigs, 1 week after a unilateral labyrinthectomy, as they were recorded in an in vitro whole brain preparation of guinea pig. The percentages of modification of the response to single-shock stimulation of the various afferent inputs of MVNn and abducens motoneurons are shown in each case, while the thickness of the pathways has been reduced or increased accordingly. Vc indicates that the modified response was induced by stimulation of the contralateral vestibular nerve. The excitatory neurons and synapses are shown in *white*, whereas the inhibitory neurons and synapses are *drawn in black*. The absence of signs inside the neurons indicates balanced levels of spontaneous activity between both sides of the brain in these compensated guinea pigs. *M* median line of the brain, *VI* abducens motoneurons

Sensory substitution is another mechanism involved on the long term by means of reweighting of extra-vestibular inputs [47, 52] (Fig. 3). The literature is rich with examples indicating that visual cues compensate for the loss of vestibular information and substitute as a reference for Earth vertical in controlling posture and trunk stability [52]. In a study performed in 50 Menière’s disease patients submitted to a curative UVN and evaluated in static posturography with eyes open or eyes closed, half of the population showed a visual strategy characterized by a better postural performance eyes open, while the second half exhibited an opposite pattern, with a better postural performance with eyes closed, suggesting a more important reliance on somatosensory inputs [53]. The role of neck information was recently investigated in alert primates using trunk rotations relative to the stationary head [54, 55]. The authors reported an increased neck sensitivity that did not increase the cervico-ocular reflex gain, suggesting a dynamic unmasking of the neck proprioceptive drive onto the ipsilesional VN complex. Interestingly, the absence of potential sensory substitution sources can lead to permanent deficits. In recent studies performed in adult Xenopus frogs submitted to unilateral labyrinthectomy at larval stages, persistent asymmetric descending vestibulo-spinal activity was observed, leading to scoliotic deformations [56, 57]. The aquatic ecophysiology and absence of body-weight-supporting limb proprioceptive signals in amphibian tadpoles had induced a permanent asymmetric motor drive to the axial musculature, as revealed by retrograde tracing of descending pathways, which showed a loss of vestibular neurons on the ipsilesional side with crossed vestibulo-spinal projections. This would provoke severe scoliotic deformations during ontogenetic development similar to the human syndrome, and might explain the detection of vestibular asymmetry in idiopathic scoliotic patients [58].

Behavioural substitution is a different recovery mechanism illustrated in vestibular patients by the pre-programming of compensatory eye saccades that substitute to the missing slow phase eye velocity of the normal VOR [18]. These authors suggest that the covert saccades could be produced by neck afferents triggered at the very start of head turn. Indeed, there is little or no recovery at all of the VOR in response to fast head movements, and the saccadic substitution helps patients to compensate and return to a normal lifestyle. In this behavioural substitution process, efferent copy signals are important. Indeed, passive, unpredictable, high acceleration head movements (vHIT) demonstrate the vestibular loss while active head movements promote the learning of new strategies and new behaviours that conceal the inadequate VOR response. Gaze stability as well as dynamic visual acuity is restored faster by active training just after the vestibular loss [59]. Other vicarious strategies to compensate the missing VOR are described in the literature. Some vestibular patients close their eyes during head movement to the lesion side, or perform blinks, two very simple strategies used to avoid retinal slip, oscillopsia and dizziness. Others move slowly their whole body as a block, and use the optokinetic reflex to stabilize gaze in the low frequency range, that is, not in the normal range of head movements. Suppression of cortical visual motion processing during head rotation is also reported as a strategy used for cancelling the perception of retinal slips [60]. Interestingly, reorganization of eye–head coordination in animal models of unilateral vestibular loss points also to different idiosyncratic strategies ([61, 62] monkey; [63] cat).

Recovery of the dynamic vestibular functions is based, therefore, on various idiosyncratic vicarious strategies, that is, on the brain orchestration of behavioural melodies taken in a repertory depending on the patients themselves. This dynamic functional reorganization implies different brain structures and neuronal networks, as shown by imaging data collected in both animal models and unilateral vestibular loss patients.

### Imaging studies in unilateral vestibular loss patients

Cerebral vestibular compensation in patients with central or peripheral unilateral vestibulopathy has largely been studied by imaging techniques in the chronic stage only when clinical signs of static vestibular imbalance have largely been resolved. Meaningful interpretation of imaging data, however, requires follow-up studies in the acute and chronic stage of vestibular lesions.

In the rat model of acute unilateral labyrinthectomy, serial FD**G**-MicroPET revealed an immediate asymmetry of cerebral regional glucose metabolism (rCGM) in the vestibular nuclei complexes and related vestibular signal processing structures (vestibulo-cerebellum, thalamus, temporoparietal cortex, hippocampus) which was re-balanced within 1 week when ataxia had improved [32]. These data suggest some imaging evidence that deafferentation-induced neural plasticity after complete unilateral vestibulopathy occurs at the level of the VN, and that VC comprises both increased somatosensory and vestibulo-cerebellar adaptation at the later stages of behavioural recovery.

In humans, follow-up brain metabolism studies (FDG-PET) comparing patients in the acute and chronic stages of peripheral vestibular imbalance due to incomplete lesions in vestibular neuritis revealed both cortical and sub-cortical activation patterns in the acute phase, which resemble those of peripheral vestibular stimulation on the contralateral side in healthy subjects [64, 65]: a contralesional increase in rCGM in posterolateral thalamus and retroinsular vestibular cortex was observed. This asymmetry reversed within 3 months with peripheral vestibular nerve recovery and behavioural restitution. VC seems to differ as the dominant ascending input is shifted from the ipsilateral to the contralateral pathways, presumably due to the missing ipsilateral vestibular input [60]. Unlike peripheral vestibular deafferentation, VC in central vestibular lesions involving the VN in lateral medullary infarctions was suspected by immediate (acute stage) hypermetabolism in the cerebellum (vermis) and brainstem (contralateral medulla comprising the VN and cerebellar peduncles), but not at cortical levels [66]. This is in accord with early brainstem commissural VC in animal lesion data [32]. After 6 months, the immediate cerebellar hypermetabolism reversed into decreased metabolism, while visual cortical areas revealed increased activity as a potential sign of sensory substitution [66].

**Fig. 4:**
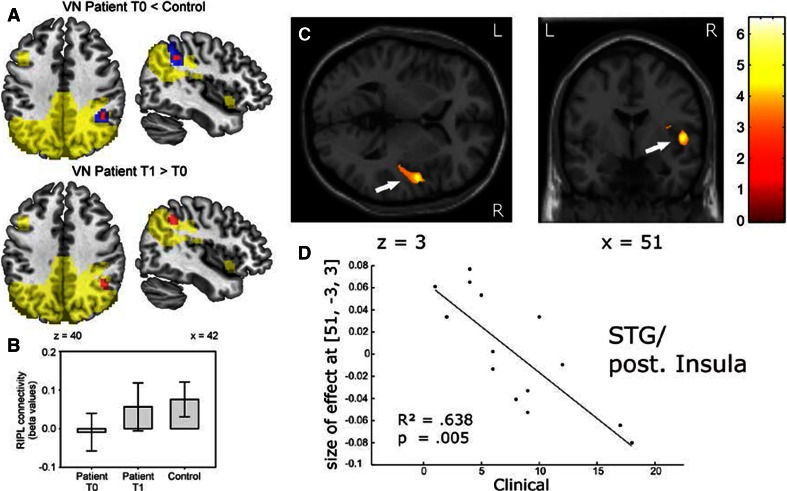
Resting-state activity and voxel-based morphometry changes in patients with unilateral vestibular loss. **a**, **b** Resting-state activity changes (independent component analysis, component 50, beta values, with standard deviation, in arbitrary units) in right intraparietal sulcus (RIPL) contrasting 20 vestibular neuritis patients at acute unilateral vestibular failure (T0) and after 3 months (T1) with control subjects. *Red area* decreased circumscribed resting-state activity in patients (*red* *p* < 0.05, corrected; *blue* *p* < 0.005, uncorrected) which is partially reversed over time. *Yellow* component mask generated across both groups (patients and controls). Modified after [67]. **c**, **d** Voxel-based morphometry in patients with unilateral vestibulopathy due to surgical resection of acoustic neuroma: Significant correlation of gray matter volume (GMV) increases (ordinates) in the superior temporal gyrus (STG)/posterior insula with the clinical vestibular score (CVS, abscissae). CVS, a score reflecting vestibular impairment on clinical examination, is shown on representative axial and coronal slices of the standard MNI template and indicated by *arrows* (*x*-, *z*-coordinates below the slices). T-scores are indicated by the coloured map. The linear regression reflects that the increase in GMV is strongest with least clinical impairment. Modified after [70]

Central VC might not only result from deafferentation but also from new functional connectivities between vestibular and multisensory brain regions. Functional connectivity of cerebral regions conveying vestibular signals has been identified in healthy subjects [67]. Distinct vestibular pathways have been delineated between the VN and the ipsilateral and contralateral (crossing at pontine and midbrain level) parieto-insular vestibular cortex (PIVC), providing potential grounds for functional VC. PIVC regions of both hemispheres are interconnected transcallosally through the anterocaudal splenium. In unilateral vestibular neuritis, resting-state activity analysis revealed decreased functional connectivity in contralateral parietal lobe (intraparietal sulcus, supramarginal gyrus), which increased over time as vestibular-induced disability declined [68], suggesting a different mode of VC at cortical level (Fig. 4a, b).

Structural changes may follow or even provoke abnormal activity or alterations in functional connectivity. Gray matter volume (GMV) changes following unilateral vestibulopathy have been found in the somatosensory cortex, multisensory vestibular cortex areas, that is, superior temporal gyrus (STG), insula, inferior parietal lobe (IPL), middle temporal areas (MT/V5), posterior hippocampus and the brainstem at the level of the gracile and VN [68–71]. Commissural fibres between the VN and at pontine levels even seemed to be increased [70]. GMV changes in the STG, posterior insula (Fig. 4c, d) and the inferior parietal lobe seem to gain clinical significance as they were related to levels of functional impairment, as corroborated by clinically assessed vestibular deficits and self-assessed vestibular impairment rating scales [69, 70]. Therefore, these structural changes may even contribute to VC. Cortical GMV increases were predominantly contralesional to peripheral vestibulopathy as a possible sign of increased use of contralateral ascending pathways due to the defective ipsilateral vestibular input [69, 70]. Structural volume increases in other sensory brain areas, i.e. somatosensory and visual cortex (motion sensitive areas MT/V5) support the sensory substitution mechanisms of adaptation following vestibular impairment [69, 70]. Over-reliance on visual information for spatial orientation is one characteristic feature of poorly recovered vestibular neuritis patients [72]. In addition, proprioceptive influence on cortical visual motion processing is enhanced in patients with chronic loss of vestibular afferent input [73], suggesting an additional cervico-visual interaction compensating for deficient vestibular input. Lines of evidence for substitution by other sensory systems come from longitudinal studies of vestibular neuritis patients which showed GMV increases during the course of 3 months in multisensory vestibular cortex, visual cortex, hippocampus and cerebellum [74]. Particularly, GMV increases in visual and cerebellar areas were related to functional recovery scores (Dizziness Handicap Inventory) reflecting a potential role of these areas in vestibular compensation [69, 70, 74].

## How to improve vestibular compensation?

The spontaneous recovery can be improved and/or accelerated, and it is not always optimal because patients can replace some lost vestibular functions (the VOR) by new maladaptive strategies (limitations of head movements). Therefore, it is important to probe the vestibular system, using the large battery of specific vestibular tests available along the compensation process [75, 76]. Two ways of VC improvement have been recently reviewed: the neuropharmacological approach [77], and the vestibular rehabilitation (VR) therapy [13, 78].

Different neurotransmitters (glutamate, acetylcholine, GABA, glycine) and neuromodulators (histamine, adrenaline, noradrenaline) regulate the vestibular functions. Drugs up-regulating or down-regulating these transmitters or their receptors may therefore alter the time course of the recovery process and the final level of compensation as well. Vestibular dysfunction activates the stress axis [79]. Steroids involved in the stress response modulate glutamatergic and GABAergic neurotransmission [36]. Other hormones such as 17β-estradiol and 5α-dihydrotestosterone can induce LTP or LTD [80]. Activation of the stress axis during VC is characterized by an up-regulation of arginine vasopressin-immunoreactive cells and corticotrophin-releasing factor-immunoreactive cells in the paraventricular nucleus, and an increase of dopamine-β-hydroxylase in the locus coeruleus of UVN cats [81]. Elevated cortisol level is found in neuro-otological diagnoses [82] and high AVP level in patients during an acute attack of Menière’s disease [83]. Cortisol and ACTH levels are positively correlated with the vestibular pathology, in Menière’s disease patients and patients with vestibular schwannoma [84]. The acute stress response is important in promoting compensatory synaptic and neuronal plasticity in the VN, but excessive stress can impair VC [85]. Poorly compensated patients often suffer of anxiety and depression [86], the reason why it is important to reduce anxiety and stress with VR therapy or anxiolytics. Up-regulation of inflammatory and neurotrophic factors in the VN can constitute a favourable environment for VC [27]. Histamine is also involved in the modulation of plasticity in the VN [87]. It inhibits GABA release through the H3 hetero-receptors, is involved in the neuron-glia cross-talk, and modulates the release of inflammatory mediators by microglial cells [88]. Histaminergic drugs can therefore accelerate the recovery process [12, 89], whereas antihistamines drugs prescribed during the acute stage can substantially reduce the neurovegetative signs and vertigo.

The VR therapy must be performed early and actively, during the time window that coincides with all the plastic reorganizations occurring in the VN and associated neuronal networks [13]. This opportunity window can be defined as a critical period during which the VC mechanisms interact with the VR therapy. The secret to get an optimal functional recovery and to regain a good quality of life is probably to take into account the extrinsic factors that characterize the sensorimotor and cognitive profile of each particular patient, and to motivate the patient. The VR physiotherapist must unlearn the maladaptive behavioural strategies and to promote the best ones, those that are adapted to day life situations. He/she must check the sensory strategies used by the patients and to adapt his/her protocol accordingly. The VR physiotherapist must reduce anxiety and stress using behavioural/cognitive therapies, and favour ecologic contexts and situations to motivate the patient. Evaluation of vestibular compensation in daily life settings seems therefore an interesting approach [90].

## Conclusions

Altogether, if one extrapolates the conclusions of the various in vivo and in vitro studies, we have summarized in animal models, we propose that VC in patients following an acute lesion would follow a “top-down” strategy. First, in the first post-lesion hours, patients would rely almost exclusively on the external cues provided by the intact sensory systems, allowing them to elaborate alternative sensorimotor strategies. We are talking here of a heavy reliance on visual and/or proprioceptive and haptic cues, and of the new, learned, vicarious idiosyncratic strategies elaborated later on. This may explain why a very active VR should occur very early after the lesion on to be optimal, because the VR interventions interact with the recovery mechanisms during the critical plastic time window of internal reorganization processes. Then, synaptic changes would occur in the activity of vestibular-related networks embedding the intact and deafferented neurons of the vestibular network. Last, modifications of the intrinsic membrane properties of first the deafferented, and then the contralesional second-order vestibular neurons would take place in a matter of few days (rodent), weeks (cat) or months (human) following the lesion.

Finally, we will quote some of the future challenges to understand VC. First, all the studies in animal models—the actual knowledge of VC—have been performed following acute lesion of the vestibular system, as it occurs in vestibular neuritis, vestibular neurotomy or traumatic injuries in patients. But the recovery mechanisms and their time course very likely differ following vestibular pathologies with slower time course such as acoustic neurinoma, Menière’s disease, or during the ageing process leading to presbyvestibulopathy, since recovery depends on the real nature (sudden or not, total or not, reversible or not: see [25, 33]) of the vestibular damage. Second, it remains poorly understood why certain species in animal models or some patients never fully compensate their vestibular deficits. Third, the miraculous drug, which would alleviate vertigo and nauseous state, and/or would promote recovery of the vestibular lesions and/or VC, remains to be found. Last but not least, VC should be evaluated in daily life settings, in ecologic conditions able to better quantify the patient’s quality of life. Vestibular Prehab, that is, pretreatment training with vestibular exercises before planned vestibular lesions, seems another interesting way to speed up the recovery of the vestibular functions (see [91]). These are exciting questions because, to our knowledge, VC is the ONLY model where causal relationships have been demonstrated between changes at the molecular/synaptic level, in neuronal networks, and the behavioural level.
